# Impact of primary tumor location in patients with RAS wild-type metastatic colon cancer treated with first-line chemotherapy plus anti-EGFR or anti-VEGF monoclonal antibodies: a retrospective multicenter study

**DOI:** 10.7150/jca.34550

**Published:** 2019-10-15

**Authors:** Antonino Grassadonia, Pietro Di Marino, Corrado Ficorella, Alessio Cortellini, Katia Cannita, Alessandro Parisi, Teresa Gamucci, Federica Zoratto, Patrizia Vici, Maddalena Barba, Ettore Porreca, Matteo Neri, Angelo Veronese, Clara Natoli, Michele De Tursi, Nicola Tinari

**Affiliations:** 1 Department of Medical, Oral & Biotechnological Sciences and CeSI-MeT, G. D'Annunzio University, Chieti-Pescara, Italy; 2 Department of Medical, Oral & Biotechnological Sciences, G. D'Annunzio University, Chieti-Pescara, Italy; 3 Medical Oncology Unit, St Salvatore Hospital, Department of Biotechnological & Applied Clinical Sciences, University of L'Aquila, Via Vetoio, 67100, L'Aquila, Italy.; 4 Medical Oncology Unit, Sandro Pertini Hospital, Rome, Italy.; 5 Medical Oncology Unit, F. Spaziani Hospital, Frosinone, Italy.; 6 Division of Medical Oncology 2, IRCCS Regina Elena National Cancer Institute, Rome, Italy.; 7 Department of Medicine and Ageing Sciences and CeSI-MeT, G. D'Annunzio University, Chieti, Italy.

**Keywords:** Metastatic colorectal cancer (mCRC), RAS wt, Tumor location, Cetuximab, Panitumumab, Bevacizumab

## Abstract

Emerging evidence supports a prognostic role of primary tumor location in metastatic colon cancer (mCC). We conducted a retrospective analysis to evaluate the effect of tumor location on prognosis and efficacy of biological agents (anti-EGFR, Cetuximab and Panitumumab, or anti-VEGF, Bevacizumab) added to first-line chemotherapy in patients with RAS wild-type (wt) mCC. Patients with newly diagnosed RAS wt mCC candidates to first-line chemotherapy with anti-EGFRs or Bevacizumab were selected. Clinical outcomes were assessed and stratified by tumor location and type of treatment. Overall, 351 patients met the inclusion criteria. Primary colon cancer was right-sided (RCC) in 105 (29.9%) patients and left-sided (LCC) in 246 (70.1%). Patients with LCC had a better OS compared to those with RCC (33.6 vs 23.5 months, HR 0.74; 95% CI, 0.55 to 0.99; p=0.049). In the overall study population, OS was not significantly different for patients treated with Cetuximab or Panitumumab as compared to those receiving Bevacizumab. However, when comparing treatment outcome according to tumor sidedness, patients with LCC treated with Cetuximab or Panitumumab had a significantly longer PFS (12.4 vs 10.7 months; HR: 0.69; 95% CI, 0.51 to 0.93; p= 0.015) and OS (40.7 vs 28.6 months; HR: 0.67; 95% CI 0.47 to 0.95; p= 0.026). No relevant differences were observed in patients with RCC.

We found evidence in support of the impact of tumor location in RAS wt mCC treated with first-line chemotherapy in association with targeted therapy. More favorable outcomes were observed in LCC patients, but not in RCC patients, treated with anti-EGFR agents compared with those who received Bevacizumab. Further, prospective and adequately sized studies are warranted to confirm our findings.

## Introduction

Colorectal cancer represents the fourth most common cancer and the third cause of cancer-related mortality worldwide 1. The wider diffusion of screening programs has largely increased the identification of precancerous lesions or cancer at an early stage of development. However, about 20% of the cases are diagnosed when the disease has spread to secondary sites, such as liver or lung 2. Moreover, following curative resection and adjuvant chemotherapy with fluoropyrimidines and oxaliplatin, the 5-year cumulative rate of recurrence has been estimated more than 25% in a recent pooled-analysis of modern-era trials 3.

In the last few decades, we have witnessed a progressive increase in median overall survival (OS), now exceeding 30 months for patients with metastatic colorectal cancer (mCRC) 4, while progression-free survival (PFS) has remained substantially unchanged. Functional to this result has been the addition of monoclonal antibodies against EGFR (Cetuximab and Panitumumab) or VEGF (Bevacizumab) to the available chemotherapy combinations as first-line treatment 5.

It was initially found that mutations located within codon 12 and 13 of exon 2 of the KRAS gene were associated with resistance to anti-EGFR monoclonal antibodies 6, 7. In addition, retrospective analyses of randomized phase III trials have shown that the addition of Cetuximab or Panitumumab to existing chemotherapy backbones improves OS only in patients with extended RAS wilde-type (RAS wt) tumors, i.e. tumors not harboring mutations in exons 2, 3 and 4 of both KRAS and NRAS 8, 9.

Since the efficacy of Bevacizumab appears to be unaffected by RAS status 10, first-line phase II and phase III trials have compared Bevacizumab and anti-EGFR agents in patients with RAS wt tumors, with mixed results 11-13. However, results from meta-analyses suggest the superiority of anti-EGFR agents over Bevacizumab in this tumor subtype 14-16.

Differential biological features have been described for right-sided colon cancer (RCC, originating from cecum, ascending colon, and proximal two-third of the transversum) and left-sided one (LCC, originating from the distal one-third of the transversum, descending colon, sigma, and rectum) 17. Rectal cancer displays some molecular peculiarities as compared to LCC 18, 19, although usually included in this group. Evidences have been provided suggesting that primary tumor location is not only prognostic, but also predictive of the efficacy of anti-EGFR agents. In fact, several retrospective studies and meta-analyses have suggested a relevant prognostic role of primary tumor location, with RCC being associated with an inferior outcome 20-33. Some studies have also suggested that the site of the primary tumor predicts for the efficacy of anti-EGFR monoclonal antibodies, with this being restricted to RAS wt LCC 28-34.

However, very few of the available studies have specifically addressed these issues in the population of patients with RAS wt metastatic tumors, which may be characterized by a more favorable prognosis as compared to the RAS-mutated ones 35. Moreover, in some cases the effect of sidedness was excluded for RAS mutated tumors 31.

With notably rare exceptions 27, 33, 34, most of these studies are retrospective analyses of randomized clinical trials, and their results are not necessarily generalizable to the population of patients encountered in the routine clinical practice.

Thus, the present multicenter retrospective study was conducted to verify the prognostic and predictive role of tumor site in a cohort of patients with RAS wt mCC treated with first-line chemotherapy plus Bevacizumab or anti-EGFR monoclonal antibodies in the real-word setting.

## Patients and Methods

### Study design and data collection

All patients with newly diagnosed mCC consecutively referred to five Italian cancer centers between January 2010 and December 2016 for first-line therapy were considered for inclusion in this study. Among them, only patients with documented RAS (KRAS/NRAS exons 2-4) or KRAS (exon 2) wild type tumors whose treatment included a biological agent (anti-EGFRs or Bevacizumab) were selected. For each patient, demographics (gender, age), baseline clinical-pathological features (tumor histotype, tumor grade, TNM stage, site of metastasis, number of metastatic sites, primary tumor location, ECOG performance status, previous adjuvant chemotherapy), and therapy-related variables (chemotherapy backbone and type of biologic agent in first-line, number of cycles, surgery for primary tumor and metastasis, chemotherapy backbones and biologic agents used as second-line) were collected. The study protocol was approved by the local Ethics Committees and informed consents were obtained from alive patients.

### Clinical Assessment

Response to treatment was based on imaging documentation, mostly CT scans, available in clinical records, and coded according to Response Evaluation Criteria in Solid Tumors (RECIST) version 1.1 36. Based on the best response, overall response rate (ORR) was defined as the proportion of patient achieving complete or partial response, while disease control rate (DCR) was defined as the proportion of those obtaining at least stability of the disease. Response and outcome measures were analyzed after stratifying patients by primary tumor location and type of biologic agent combined to first-line chemotherapy.

Progression-free survival (PFS) was calculated from initiation of first-line therapy to disease progression or death (whichever occurred first). Overall survival (OS) was defined as the time from therapy initiation to death or last annotation on clinical records. Patients with rectal cancer were excluded from the study. The date of study cutoff was December 15, 2018.

### Statistical Analysis

Standard descriptive statistics were used to summarize patients' characteristics. Median values and ranges were used for continuous variables, while percentages were computed for categorical variables. Proportions were compared by Pearson's chi-square test or Fisher's exact test, depending on the size and number of the groups compared. Survival analyses were performed according to the Kaplan-Meier method and differences between curves tested by log-rank. Survival curves were truncated at 60 months, since the number of patients remaining at risk was too small afterward. Univariate Cox regression analysis was used to estimate hazard ratio (HR) and 95% confidence intervals (95%CI) for PFS and OS. A p value ≤ 0.05 was retained as the limit of statistical significance. The SPSS version 15.0 statistical software was used to perform all the analyses.

## Results

### Study population and baseline characteristics

**Figure 1:**
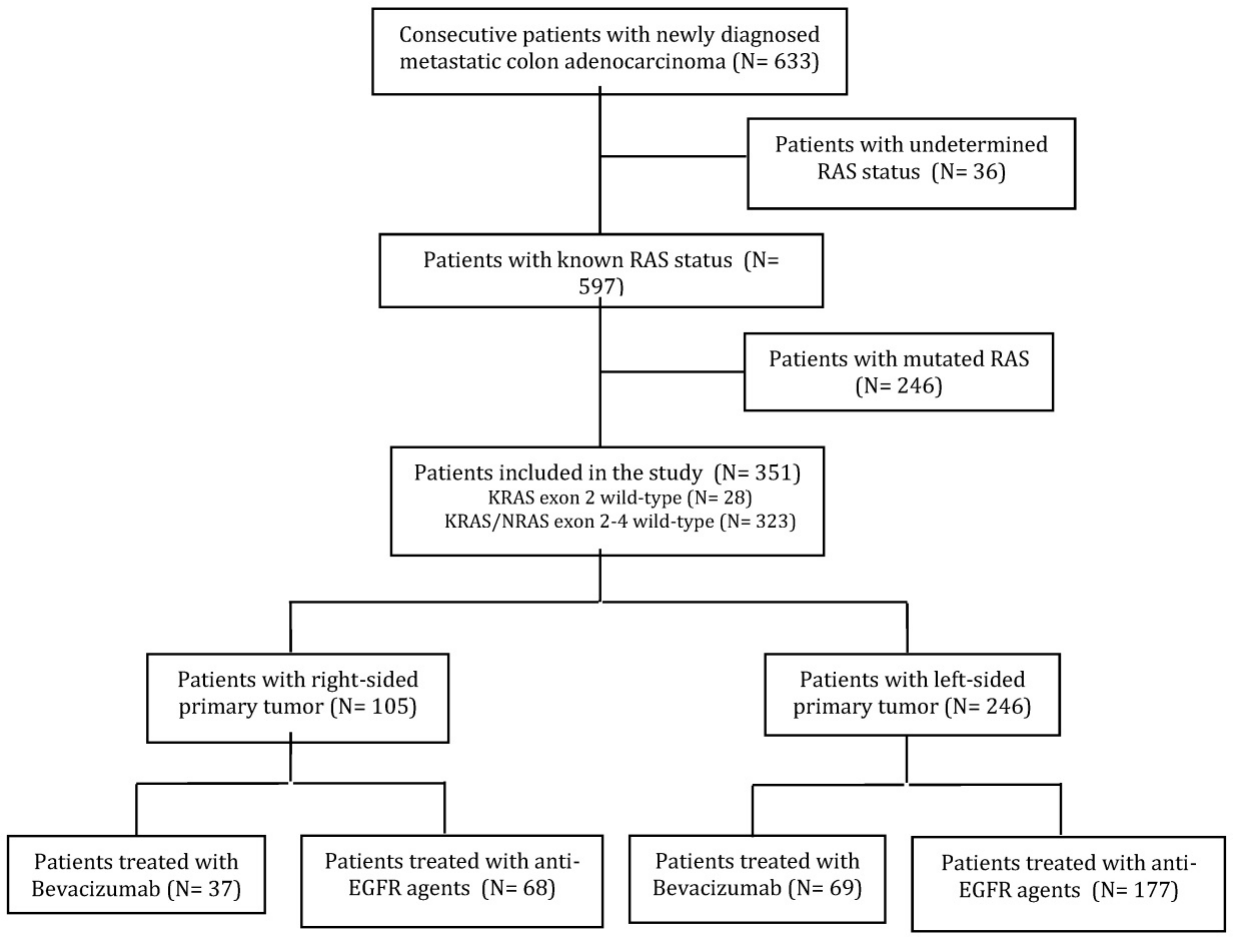
CONSORT diagram.

Six hundred and thirty-three consecutive patients with newly diagnosed mCC were treated with first-line therapy at the participating Institutions. Thirty-six patients with undetermined KRAS or RAS status and treated only with chemotherapy were excluded from the study, as were 246 patients with mutations in exons 2-4 of KRAS or NRAS. Overall, 351 patients met the inclusion criteria (Figure 1). Among them, for 28 patients (8%) the RAS wild-type status was determined by analyzing KRAS exon 2 only. The majority of patients were male (61.5%) and median age was 65 years. The primary tumor was right-sided (including cecum, ascending and transverse colon) in 105 (29.9%) patients, left-sided (descending colon and sigma) in the remaining 246 (70.1%).

One hundred and five patients (29.9%) were previously treated with fluoropyrimidine-based adjuvant therapy, which included oxaliplatin in most cases (65.7%). At diagnosis, 168 (49.7%) patients presented with multiple site metastases, mostly localized in liver and lungs. Two-hundred and forty-five (69.8%) patients received an anti-EGFR agent (Cetuximab in 154 and Panitumumab in 91 cases), and 106 (30.2%) were treated with Bevacizumab. Fluorouracil, folinic acid, and irinotecan (FOLFIRI) was the most widely used chemotherapy backbone (41.3%), especially in association with Cetuximab (88.5% of the cases).

Main patients' characteristics according to the location of primary tumor are summarized in Table 1. With the exception of a higher prevalence of subjects older than 65 among those with RCC (p= 0.026), baseline and treatment characteristics were well balanced between the two groups. Patients with LCC were prevalently treated with an anti-EGFR agent as compared to those with RCC (71.95 vs 64.8%, respectively), although the difference was not statistically significant (p= 0.179). Patients receiving maintenance therapy were 81 among those receiving anti-EGFRs and 37 among those treated with Bevacizumab.

One hundred and forty (57.1%) patients among those treated with Cetuximab or Panitumumab, and 74 (69.8%) among those treated with Bevacizumab received at least one additional line of treatment after disease progression. Second-line therapy included Bevacizumab in 80% of the patients treated with anti-EGFRs in first-line, while a similar percentage (79.8%) of patients receiving Bevacizumab in first-line was treated with an anti-EGFR agent at progression. Notably, the biological agent used in first-line was maintained beyond progression in 14.3% of patients in the anti-EGFR group, and in 20.3% of those treated with Bevacizumab.

### Response and Disease Control Rate

In the whole cohort of patients, analysis of best response revealed 30 (8.5%) complete responses, 152 (43.3%) partial responses and 97 (27.1%) stable diseases. Overall response rate was significantly higher in the group of patients with LCC as compared to the RCC group (56.9% vs 40.0 %; p= 0.004), as was DCR (81.3% vs 65.7%; p=0.002).

When the type of biologic agent was considered, neither ORR (54.3% vs 46.2%) nor DCR (77.1% vs 75.5%) were significantly different between patients treated with anti-EGFRs or Bevacizumab. Similarly, no difference in ORR and DCR was observed between treatment groups in LCC or RCC. Overall, 95 patients underwent surgical removal of metastatic lesions, mostly after first-line therapy. More frequently, although not significantly, they had left-sided tumors (28.9% vs 22.8%).

### Progression-Free Survival and Overall Survival

**Figure 2:**
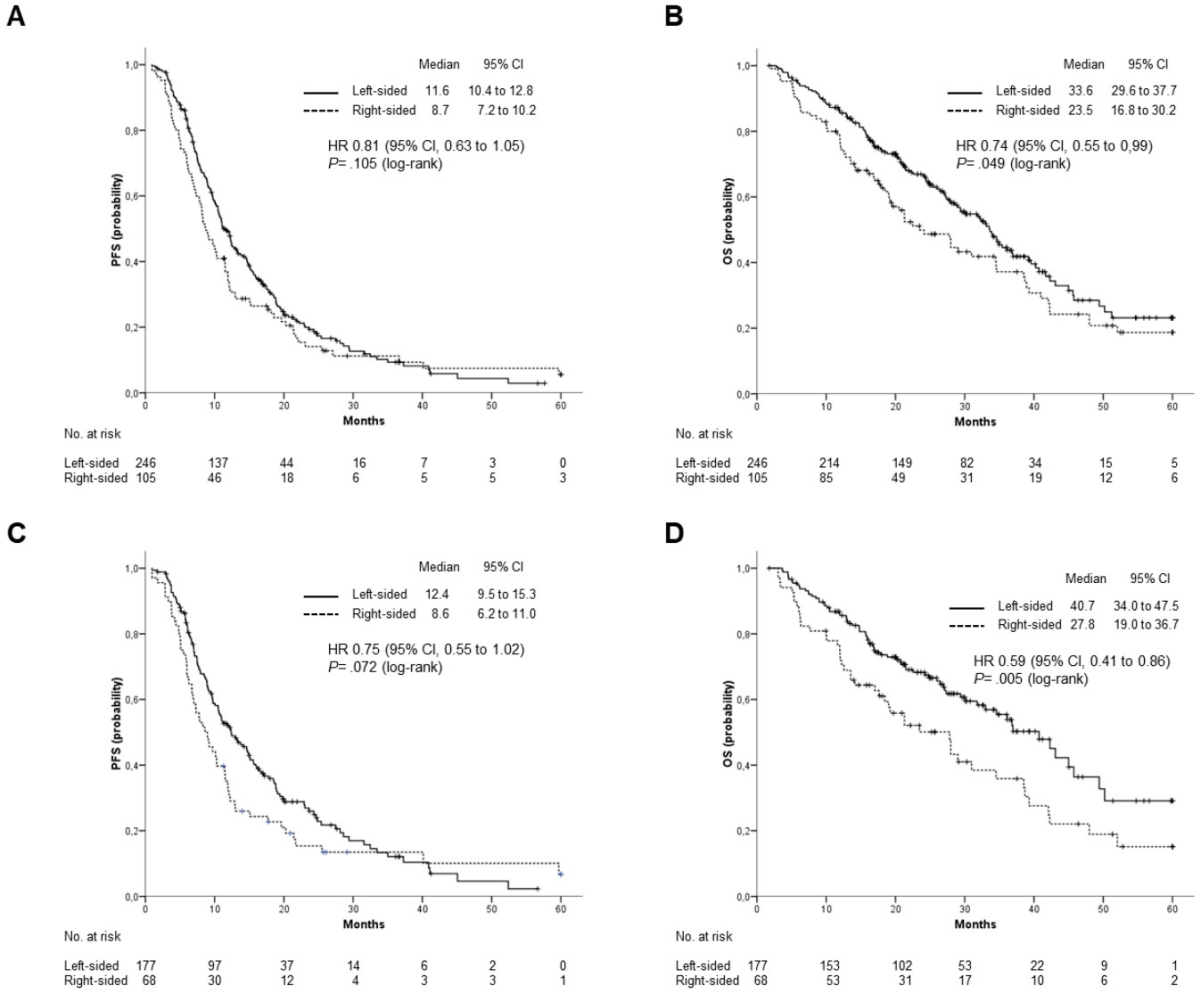
** Outcomes stratified by primary tumor location**. (A), PFS and (B), OS in the overall population; (C), PFS and (D), OS in patients treated with anti-EGFR (Cetuximab or Panitumumab).

At the date of study cutoff, 193 (55%) patients were dead. Median follow-up of surviving patients was 26.9 months (interquartile range 18.1 to 38.1 months). Overall survival was significantly, although marginally, affected by primary tumor location. Median OS was 33.6 months for patients with LCC and 23.5 months for those with a RCC (HR: 0.74; 95% CI, 0.55 to 0.99; p=0.049). A trend toward superior PFS was also observed for patients with LCC, but the difference was not statistically significant (11.6 vs 8.7 months; HR: 0.81; 95% CI: 0.63 to 1.05; p= 0.11) (Figure 2 A and B). Median OS was numerically, but not significantly, different for patients treated with Cetuximab or Panitumumab as compared to those receiving Bevacizumab (34.6 vs 28.1 months; HR: 0.81; 95% CI, 0.65 to 1.08; p= 0.16). Marginally significant was the difference in PFS (11 vs 10.3 months; HR: 0.78; 95% CI, 0.61 to 0.99; p= 0.045). Anti-EGFR containing regimens were more effective in patients with LCC as compared to those with RCC. Indeed, median OS was significantly higher among patients included in the former group (median OS: 40.73 vs 27.83 months; HR: 0.59; 95% CI: 0.41 to 0.86; p= 0.005), while a trend toward statistical significance was observed for PFS (median PFS: 12.4 vs 8.63; HR:0.75; 95% CI: 0.55 to 1.02; p= 0.072) (Figure 2 C and D).

On the contrary, efficacy of Bevacizumab containing regimens was independent from tumor location. Median OS was 28.6 and 22.5 months for patients with LCC and RCC, respectively (HR: 1.07; 95% CI: 0.66 to 1.75; p=0.784), while median PFS was 10.7 vs 8.7 months (HR: 1.06; 95% CI: 0.69 to 1.62; p=0.797).

**Figure 3:**
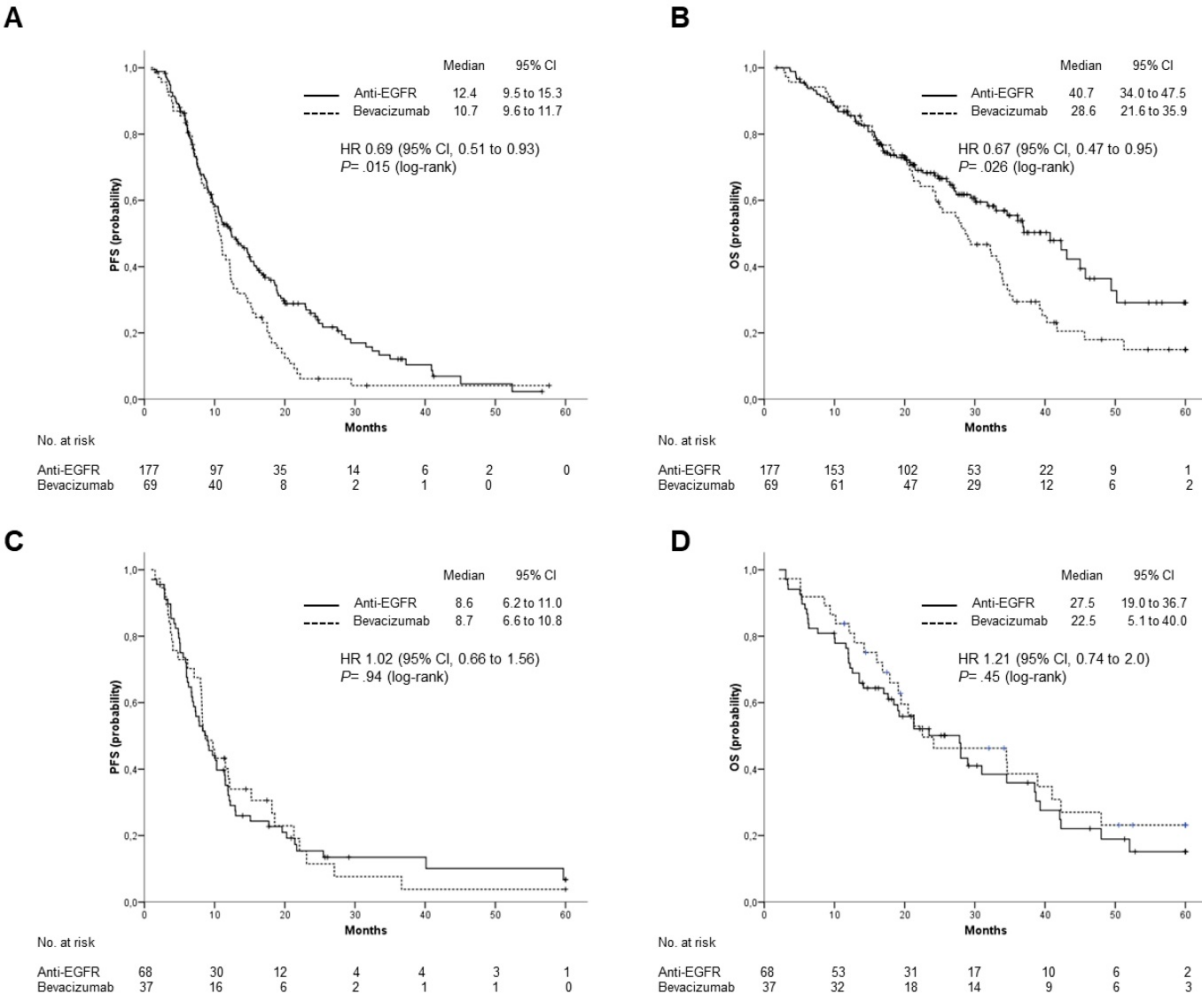
** Outcomes stratified by type of biological treatment, anti-EGFR (Cetuximab or Panitumumab) vs Bevacizumab**. (A), PFS and (B), OS in patients with left-sided primary tumor; (C), PFS and (D), OS in patients with right-sided primary tumor.

When the efficacy of anti-EGFR agents versus Bevacizumab was analyzed according to tumor location, patients with LCC treated with Cetuximab or Panitumumab had a significantly longer median OS (40.7 vs 28.6 months; HR: 0.67; 95% CI 0.47 to 0.95; p= 0.026) and PFS (12.4 vs 10.7 months; HR: 0.69; 95% CI, 0.51 to 0.93; p= 0.015) (Figure 3 A and B). For patients with RCC, neither OS (median OS: 27.5 vs 22.5 months; HR: 1.21; 95% CI: 0.74 to 2.0; p=0.45) nor PFS (median PFS: 8.63 vs 8.7 months; HR: 1.02; 95% CI, 0.66 to 1.56; p= 0.94) were significantly different between patients treated with anti-EGFRs and those treated with Bevacizumab (Figure 3 C and D).

## Discussion

In the present retrospective study, we sought to assess the relevance of primary tumor location in a cohort of consecutive patients with RAS wt metastatic colon cancer treated with first-line chemotherapy plus an anti-EGFR agent or Bevacizumab at five Italian institutions outside of randomized clinical trials.

First of all, our analysis suggests that patients with LCC, regardless of the type of treatment received, have superior ORR, DCR and OS, with only a trend toward a better PFS. Overall, the differences we found for ORR (56.9% vs 40%), DCR (81.3% vs 65.7%), median OS (33.6 vs 23.5 months) and median PFS (11.6 vs 8.7 months) appear to be in line with those reported in previous studies. For example, post-hoc analyses of the CALGB/SWOG 80405 phase III trial of first-line FOLFIRI plus Cetuximab or Bevacizumab in patients with RAS wt mCRC, found that RCC is associated with a substantially reduced median OS (19.4 vs 34.2 months) as compared to LCC, which translate in about 40% increase in risk of death 28.

Similar findings were reported in a pooled analysis of five randomized trials of first-line treatment and one randomized trial of second-line treatment 30. In this case, as in our study, the negative prognostic impact of the right location was reported also for PFS and ORR 30. Karman et al. also reported an improved OS for patients with RAS wt LCC treated in routine clinical practice 27. In this study, median OS for patients with left- and right-sided tumors, as gathered from the published survival curves (about 55 vs 31 months), was substantially higher than those reported by us. However, it should be noted, that this cohort is quite different from ours, being composed of younger patients (median age 56) with rectal cancer in about 60% of the cases.

Another real-life retrospective study focusing on RAS wt mCRC has also found a superior OS for LCC as compared to RCC (42 vs 37 months), but this difference was not statistically significant likely due to the small number of patients, as acknowledged by the Authors 37. ORR was reported to be identical between the two groups, but complete response was more frequently, although not significantly, observed among patients with RCC 37.

In our series, PFS and OS, the latter only numerically, were higher in patients treated with anti-EGFRs as compared to those receiving Bevacizumab, while no significant difference in response was observed. Although not significantly different from a statistical viewpoint, median OS values for left- versus right-sided tumors (34.6 vs 28.1 months) appear to be very similar to those reported for patients treated with FOLFIRI plus Cetuximab or Bevacizumab in the post-hoc analyses of the FIRE-3 trial restricted to the RAS wt subgroup of patients, in which, however, no difference in PFS was seen 4.

Superior median OS (41.3 vs 28.9 months) for patients treated with Fluorouracil, folinic acid, and Oxaliplatin (FOLFOX) plus Panitumumab as compared to those receiving FOLFOX plus Bevacizumab, have been reported also in the subgroup of RAS wt patients enrolled in the PEAK randomized phase II trial 13. In this case, median PFS for the two treatment groups (13 vs 9.5 months) were similar to those observed in our study. On the contrary, our results are not consistent with those of CALGB/SWOG 80405 phase III trial, in which no difference in OS, PFS and ORR was observed between treatment groups 28.

Among RAS wt patients treated with anti-EGFR agents, we observed a significantly superior OS for those with primary tumor located on the lift side, with a trend toward superiority for PFS. Similarly, in the retrospective analyses of the CRYSTAL and FIRE-3 29, AIO KRK-0104 trial 31, and CALGB/SWOG 80405 trial 28 higher OS and PFS were found among patients with LCC treated with anti-EGFR agents. A superior OS for patients with LCC treated with Panitumumab or Cetuximab has also been reported by Arnold et al. in their pooled analysis of randomized trials 30. Finally, the use of Cetuximab was associated with longer OS and PFS for patients with LCC as compared to those with RCC in an oncology community-based retrospective study 34.

The fact that, differently from what we observed for anti-EGFR antibodies, the efficacy of Bevacizumab was not influenced by primary tumor location, may possibly indicate a predictive effect of primary tumor location. Indeed, when efficacy of anti-EGFR agents was compared to that of Bevacizumab after stratification by site of primary tumor, we found that the former were associated with a significant advantage in median OS (40.7 vs 28.6 months) and PFS (12.4 vs 10.7) in the group of patients with LCC, while no difference was observed in those with RCC.

A comparison of the two biological types of RAS wt tumors according to sidedness has been reported in retrospective analyses of FIRE-3 29, PEAK 32, CALGB SWOG 80405 28 and in a large population-based retrospective study 33. In all these studies, as in ours, survival was higher for the left-sided group of patients when treated with the anti-EGFR monoclonal antibody as compared with Bevacizumab. On the contrary, while for FIRE-3 and PEAK no difference in efficacy between the two types of treatment was reported for right-sided tumors, as in our study, for the CALGB SWOG 80405 trial and the population-based study a significant advantage for Bevacizumab has been observed 28, 33.

Reasons for the discrepancies among these studies are largely speculative. However, it has been suggested that the more frequent use of Bevacizumab beyond progression in the CALGB trial as compared to FIRE-3 may give a survival advantage to patients treated with this monoclonal antibody in first-line, thus explaining their higher survival observed in the former study 38. The recently published results of a phase II trial, in which patients with KRAS wt mCRC progressing after first-line Bevacizumab plus chemotherapy were randomized to maintain the same biological agents or to cross-over to Cetuximab, provide arguments supporting this hypothesis 39. Interestingly, in our series only about 20% of patients maintained Bevacizumab beyond progression, which may explain why findings are closer to those of FIRE-3 trial.

It is believed that the prognostic and predictive effect associated with the site of the primary tumor descend from distinctive biological characteristics, rather than simply from the different embryological origin of the right and left side of the colon. From a molecular view point, mutations in the APC, SMAD4, TP53, and KRAS genes are more frequently described for LCC 40. In addition, LCC presents higher chromosomal instability, amplification of the epidermal growth factor receptor and overexpression of its ligands epiregulin and amphiregulin 41. On the other hand, RCC more often displays a high CpG island hypermetilation phenotype (CIMP-high), high microsatellite instability (MSI-high) 42, a higher incidence of RAS and PI3K mutations 43, and BRAF mutations 41.

For its retrospective nature, our study has some limitations. In a small group of patients (8%), testing of RAS status was limited to KRAS exon 2. Therefore, it cannot be excluded that some RAS mutant cases have been misclassified as wild-type. In addition, all cases with a tumor in the transverse colon were included in the right-sided group, since from the clinical records it was not possible to distinguish those originating in the distal third of transverse colon that should have been included in the left-side-group. It should also be noted that the study did not account for the possible different policy adopted for the timing of response assessment at the participating institutions, which may have affected estimation of PFS. Moreover, it was not possible to analyze the cancer-specific survival because the cause of death was not always found in the clinical records. Assessment of BRAF status is not usually performed in routine clinical practice, and its unavailability surely represents one of the main limitations of our study. Given that BRAF mutation prevents response to anti-EGFR monoclonal antibodies 44, this might have led to an underestimation of the effect of anti-EGFR agents, especially in RCC patients. Finally, it should be acknowledged that the limited size of our cohort dictates caution in the interpretation of some subgroup analysis.

Nevertheless, our study is one of the few real-life studies that have specifically addressed the prognostic and predictive role of tumor sidedness in RAS wt mCRC patients treated in first-line with a combination of chemotherapy plus anti-EGFR agents or Bevacizumab.

## Conclusion

Our findings support the notion that in this population of patients from the real-world setting response to anti-EGFR agents and prognosis are significantly influenced by tumor sidedness. In particular, we observed significantly more favorable outcomes following administration of Cetuximab or Panitumumab compared with Bevacizumab in combination with chemotherapy for the treatment of LCC. Conversely, no significant differences emerged in patients' outcomes by category of biological agents for RCC.

## Figures and Tables

**Table 1:**
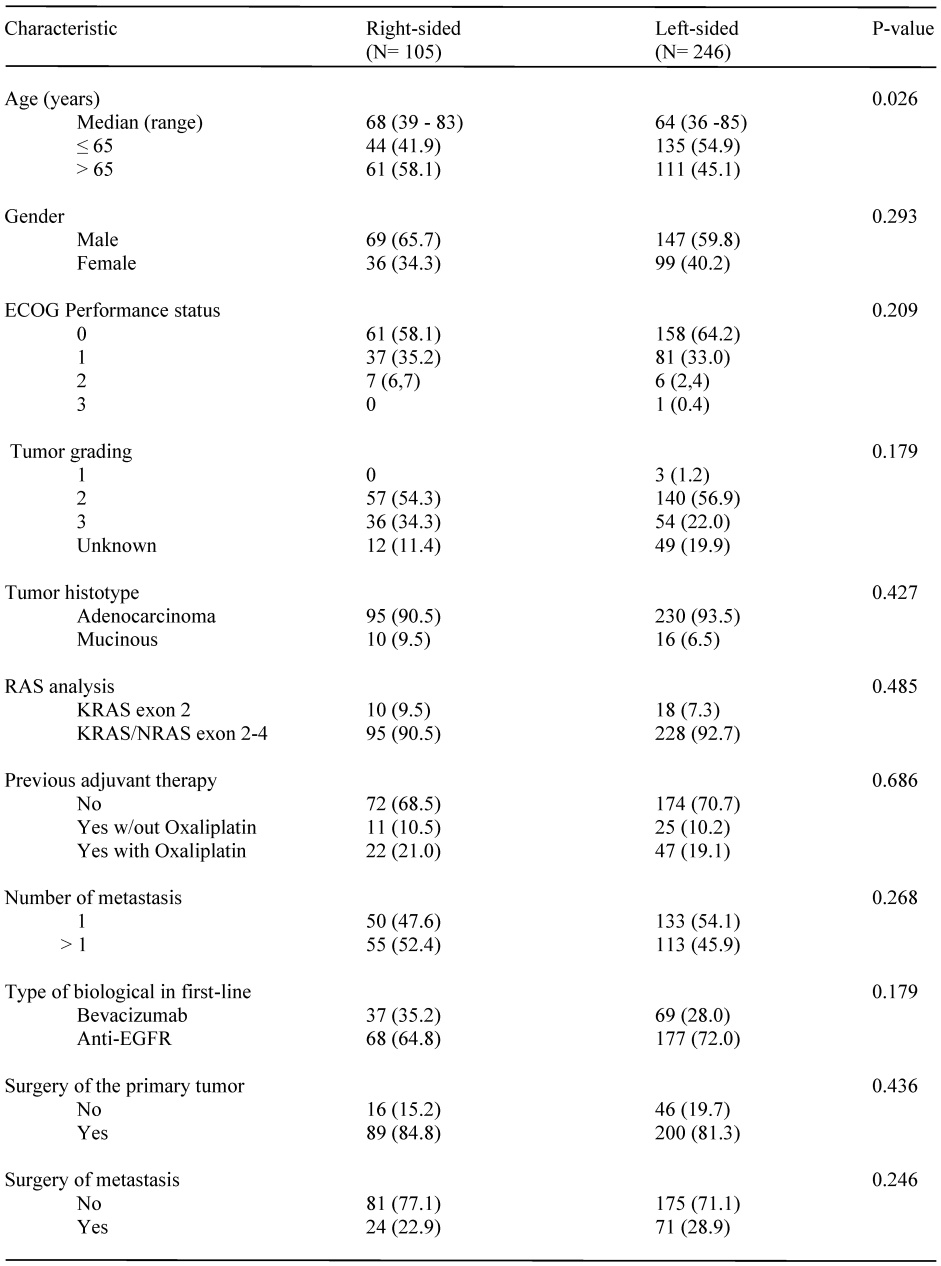
Patients characteristics and treatments by side of primary tumor
